# Carcinoma ex pleomorphic adenoma of the upper lip: a case of an unusual malignant component of squamous cell carcinoma

**DOI:** 10.1186/1477-7819-11-234

**Published:** 2013-09-17

**Authors:** Eiji Mitate, Shintaro Kawano, Tamotsu Kiyoshima, Toshiyuki Kawazu, Toru Chikui, Yuichi Goto, Ryota Matsubara, Seiji Nakamura

**Affiliations:** 1 Section of Oral and Maxillofacial Oncology, Division of Maxillofacial Diagnostic and Surgical Sciences, Faculty of Dental Science, Kyushu University, 3-1-1, Maidashi, Higashi-ku, Fukuoka 812-8582, Japan; 2 Laboratory of Oral Pathology, Division of Maxillofacial Diagnostic and Surgical Sciences, Faculty of Dental Science, Kyushu University, 3-1-1, Maidashi, Higashi-ku, Fukuoka 812-8582, Japan; 3 Section of Oral and Maxillofacial Radiology, Division of Maxillofacial Diagnostic and Surgical Sciences, Faculty of Dental Science, Kyushu University, 3-1-1, Maidashi, Higashi-ku, Fukuoka 812-8582, Japan

**Keywords:** Carcinoma ex pleomorphic adenoma, Squamous cell carcinoma, Minor salivary gland

## Abstract

**Background:**

Squamous cell carcinoma (SCC) as the malignant component of carcinoma ex pleomorphic adenoma (CXPA) occurring in upper lip is rare.

**Case report:**

A 55-year-old male patient presented with an asymptomatic mass of the upper lip that had noticed 8 years previously. The mass was clinically suspected to be a benign salivary gland tumor based on palpation and magnetic resonance imaging findings. A needle biopsy was then carried out, and the pathological diagnosis was pleomorphic adenoma. The tumor was removed under general anesthesia. Histopathological examination revealed well-demarcated tumor tissues showing typical histologic features of pleomorphic adenoma. However, SCC tissue with several mitotic figures was found in the central area of the tumor tissue. The tumor was finally diagnosed as CXPA. There was no evidence of recurrence or metastasis 6 years postoperatively.

**Conclusion:**

This is the first report of CXPA of the upper lip with an unusual malignant component of SCC.

## Background

Carcinoma ex pleomorphic adenoma (CXPA), described as a rare malignant epithelial tumor arising from a pre-existing pleomorphic adenoma (PA), accounts for approximately 12% of all salivary gland malignancies and 6.2% of all PAs [1]. CXPA most frequently arises in the parotid gland. However, it may also originate from the submandibular glands and minor salivary glands, most commonly those in the palate and rarely those in the upper lip [2]. The typical history is that of a chronic mass present much longer than 3 years, with rapid growth over the previous few months. Histopathologically, the malignant component is most frequently a poorly differentiated adenocarcinoma or undifferentiated carcinoma, but uncommonly is a squamous cell carcinoma (SCC) [1]. We herein present an extremely rare case of CXPA of the upper lip with an unusual malignant component of SCC.

## Case presentation

**Figure 1:**
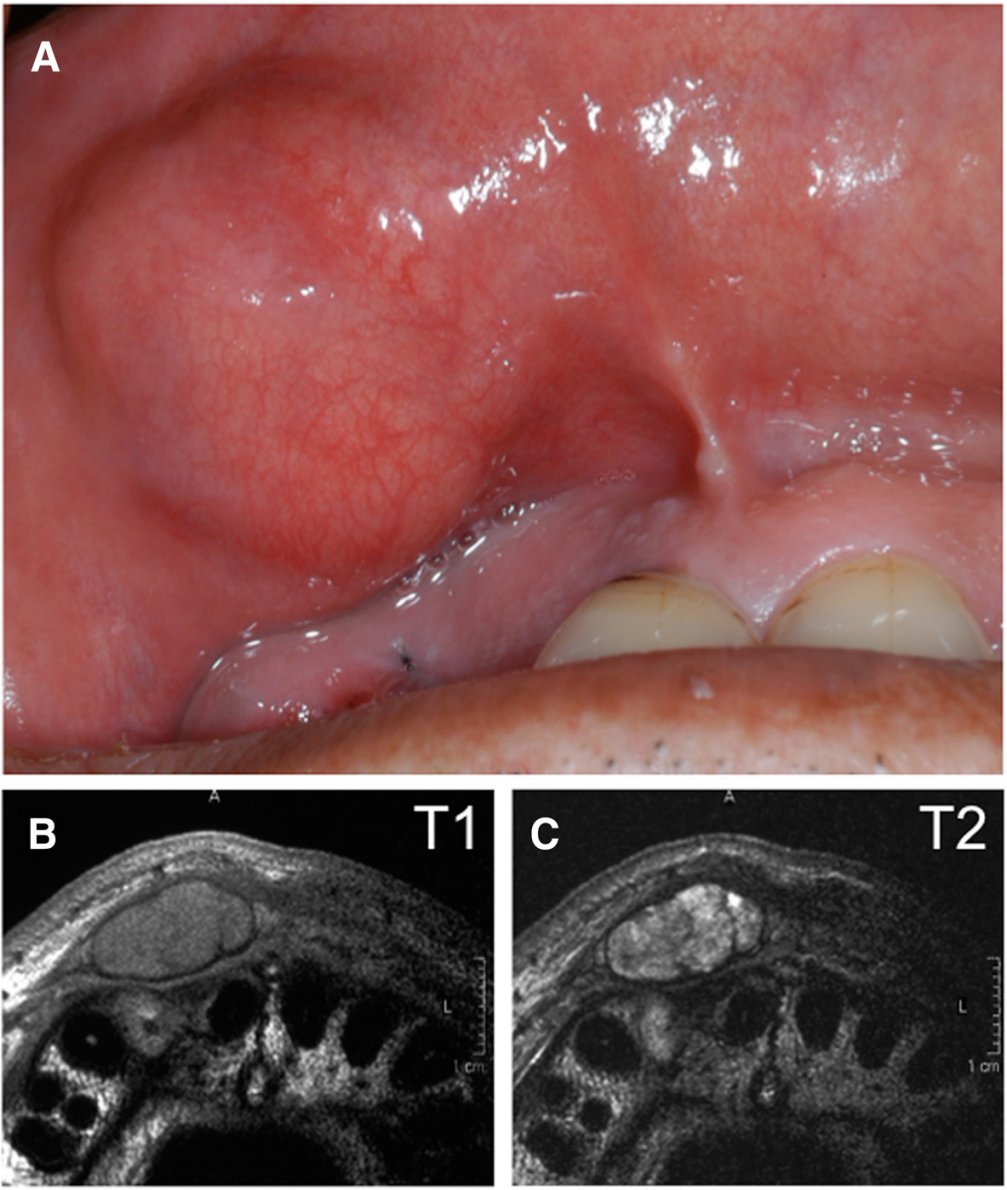
**Intraoral findings and magnetic resonance images. (A)** An elastic hard mass is located in the right upper lip. Magnetic resonance images show an apparently circumscribed mass lesion in the right upper lip. **(B)** T1-weighted image. **(C)** T2-weighted image.

**Figure 2:**
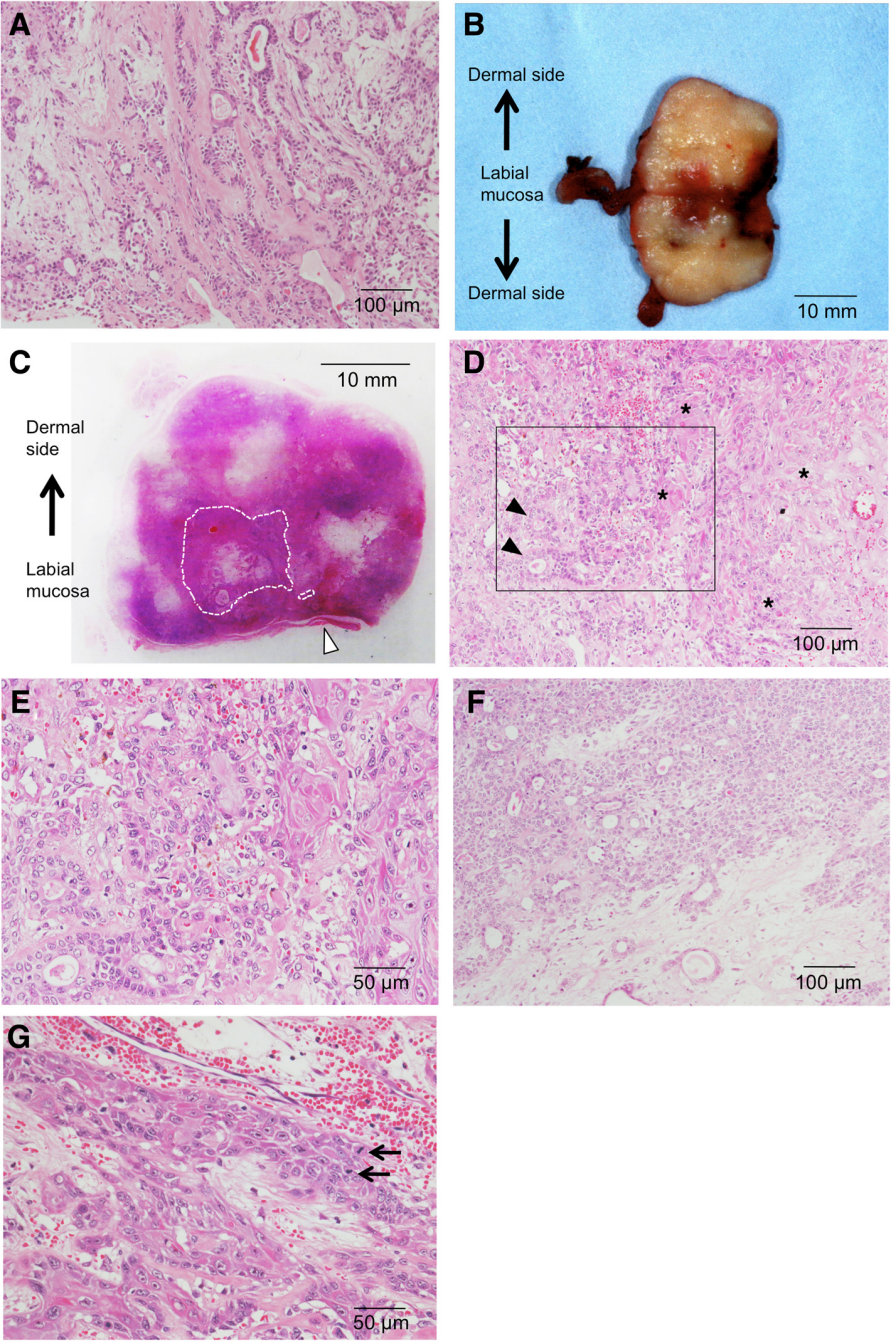
**Resected specimens and histopathological findings (hematoxylin and eosin stain). (A)** The specimen obtained by needle biopsy shows typical histopathologic features of pleomorphic adenoma (PA). Bar = 100 μm. **(B)** The cut surface of the excised tumor is glistening, heterogeneous, and tan. Bar = 10 mm. **(C)** The loupe image shows that the tumor is completely encapsulated. The malignant component is located in the central area of the tumor. The arrowhead shows the labial mucosa. The dotted lines indicate the squamous cell carcinoma (SCC) component. Bar = 10 mm. **(D)** The unclear borderline between the PA and SCC components at low magnification. Arrowheads and asterisks show ductal structures and well-differentiated squamous cell component, respectively. Bar = 100 μm. **(E)** High magnification of Figure 2D. Bar = 50 μm. **(F)** The PA region. Bar = 100 μm. **(G)** The SCC region. Arrows show mitotic figure of cancer cells. Bar = 50 μm.

A 55-year-old male patient presented with an asymptomatic mass of the upper lip that he had noticed 8 years previously. His past medical history included depression. The patient’s systemic findings were a good constitution and nourished condition. Intraoral examination revealed a painless, apparently circumscribed, movable, elastic hard, and little-finger-sized tumor mass in the upper lip (Figure 1A). No sensory or motor paralysis was observed in the maxillofacial region. No abnormal findings were observed in the bilateral cervical lymph nodes. Gadolinium-enhanced T1-weighted magnetic resonance imaging showed that the tumor mass had clear boundaries and low-signal intensity (Figure 1B). On T2-weighted images, the mass showed slight high-signal intensity with internal heterogeneity (Figure 1C). These findings suggested the presence of a benign tumor in the upper lip. Needle biopsy was thus carried out, and the pathological diagnosis was PA (Figure 2A). The tumor was removed under general anesthesia. Grossly, the cut surface of the excised tumor was glistening, heterogeneous, and tan (Figure 2B). Histopathological examination revealed typical histologic features of PA; the tumor was well demarcated and completely encapsulated (Figure 2C). However, a well-differentiated SCC component was found in the central region of the tumor. The tumor was finally diagnosed as CXPA. There was no focal capsular invasion of malignant cells or infiltration into the adjacent normal tissue. We thus chose a wait-and-see approach without postoperative treatment. There was no evidence of recurrence or metastasis for 6 years after the operation.

### Pathologic findings

The specimen obtained by needle biopsy showed a proliferation of polygonal epithelial and myoepithelial cells in myxomatous, fibrous, and hyaline backgrounds. Typical bilayered ductal structures comprising these cells were observed. These histological features are usually noted in PA (Figure 2A). Meanwhile, the sample removed during the operation revealed that the well-differentiated SCC component was located in the central region of the tumor (Figure 2C). However, no extracapsular invasion of malignant cells into the adjacent normal tissue was found. The border between the PA and SCC was unclear and intermingled (Figure 2D,E). In the PA region, the round and ovoid epithelial cells showed a compact cellular arrangement in the form of a large sheet with myxoid and fibrous stromata (Figure 2F). In the SCC region, cancer cells with several mitotic figures showed invasive growth and individual cell keratinization (Figure 2G).

## Discussion

Malignant derivatives of PA of salivary gland origin are rare and include three types. CXPA is the most common malignant change, whereas carcinosarcoma and metastasizing PA are less common. CXPA is further subclassified into a noninvasive type, minimally invasive type (≤1.5 mm penetration of the malignant component into the extracapsular tissue), and invasive type (>1.5 mm invasion from the tumor capsule into adjacent tissues) [3]. Noninvasive CXPA is also referred to as carcinoma *in situ* arising in PA. The prognoses of first two types are usually favorable, while the latter has a guarded prognosis. In the present case, the CXPA was a noninvasive type because the tumor was completely encapsulated and extracapsular spreading of the malignant component was not histopathologically seen. The malignant component of CXPA is most frequently a poorly differentiated adenocarcinoma or undifferentiated carcinoma [3]. Seifert and colleagues [4] reported 38 cases of CXPA, among which SCC accounted for 10% of the histologic variety of malignant components. Furthermore, in 73 cases of CXPA reported by Lewis and colleagues [5] and 37 cases of CXPA reported by Tortoledo and colleagues [6], no malignant components were classified as SCC. SCC as the malignant component of CXPA thus seems to be rare; only a few such case reports are present in the English language literature [7-10]. In the present case, the SCC nests showed no continuity with the labial mucosa, and extracapsular invasion was not observed. Taken together, these findings suggest that the SCC originated from the epithelial component of the PA.

CXPA in the minor salivary glands commonly occurs in the palatal region, although in rare cases it can be found in the lip [2]. Therefore, from the point of view of the tumor origin and pathological features, this case seems to be extremely rare. It is the first report of CXPA of the upper lip with an unusual malignant component of SCC. Lewis and colleagues [5] reported that all patients with intracapsular carcinoma were alive and well without evidence of disease progression. However, CXPA generally displays aggressive and destructive behavior and has high recurrence and metastatic rates. Careful follow-up is thus necessary, although there has been no evidence of recurrence or metastasis in the present case.

## Conclusion

This is the first report of CXPA of the upper lip with an unusual malignant component of SCC. The SCC originated from the epithelial component of the PA.

## Consent

Written informed consent was obtained from the patient for publication of this case report and any accompanying images. A copy of the written consent is available for review by the Editor-in-Chief of this journal.

## Abbreviations

CXPA: carcinoma ex pleomorphic adenoma; PA: pleomorphic adenoma; SCC: Squamous cell carcinoma.

## Competing interests

The authors declare that they have no competing interests.

## Authors’ contributions

SK designed and EM drafted the manuscript; EM, SK, TKi, TKa, TC, YG and RM were involved in the conception of the report and acquisition of data; SK and SN revised the manuscript and supervised. All authors read and approved the final manuscript.
